# 4,4′-DMAR *in-vivo* acute cardiotoxicity: differences between (±)*cis*-4,4′-DMAR and its coadministration with the (±)*trans*-4,4′-DMAR isomer

**DOI:** 10.3389/fcvm.2026.1763456

**Published:** 2026-06-16

**Authors:** Giorgia Corli, Elisa Roda, Beatrice Marchetti, Micaela Tirri, Sabrine Bilel, Marta Bassi, Beatrice Benedetti, Elena Cavarretta, Giacomo Frati, Fabrizio De Luca, Carlo Alessandro Locatelli, Fabio De-Giorgio, Matteo Marti

**Affiliations:** 1 Department of Translational Medicine, Section of Legal Medicine, LTTA Center and University Center of Gender Medicine, University of Ferrara, Ferrara, Italy; 2 Laboratory of Clinical & Experimental Toxicology, Pavia Poison Centre, National Toxicology Information Centre, Toxicology Unit, Istituti Clinici Scientifici Maugeri IRCCS Pavia, Pavia, Italy; 3 Section of Legal Medicine, Department of Health Care Surveillance and Bioetics, Università Cattolica del Sacro Cuore, Rome, Italy; 4 Fondazione Policlinico Universitario A. Gemelli IRCCS, Rome, Italy; 5 Department of Medical-Surgical Sciences and Biotechnologies, Sapienza University of Rome, Latina, Italy; 6 Advanced Cardiovascular Therapies Unit, Bambino Gesù Children’s Hospital, IRCCS, Rome, Italy; 7 IRCCS, Neuromed, Pozzilli, IS, Italy; 8 Department of Veterinary Medicine and Animal Sciences (DIVAS), University of Milan, Lodi, Italy; 9 Collaborative Center for the Italian National Early Warning System, Department of Anti-Drug Policies, Presidency of the Council of Ministers, Rome, Italy

**Keywords:** 4,4′-DMAR, arrhythmia, cardiotoxicity, NPS, stimulant

## Abstract

**Backgrounds/aims:**

Synthetic stimulants represent the largest group of new psychoactive substances and include the aminorex class, which possess anorectic properties and a pharmacological profile similar to that of amphetamine. Among these, 4,4′-dimethylaminorex (4,4′-DMAR) is one of the best-known synthetic stimulants with a chiral structure, which gives rise to two stereoisomers [(±)*cis*-4,4′-DMAR and (±)*trans*-4,4′-DMAR] with distinct pharmacological and toxicological characteristics. This substance has been detected in cases of intoxication and deaths among adults and adolescents. Although 4,4′-DMAR can also induce respiratory impairments, tachycardia, and sudden cardiac death/arrest, clinical and preclinical investigations of its cardiorespiratory toxicity remain limited. Thus, this study aimed to investigate the cardiorespiratory effects of (±)*cis*-4,4′-DMAR (1, 3, and 10 mg/kg) administration and the coadministration of 1 mg/kg (±)*cis*-4,4′-DMAR and 30 mg/kg (±)*trans*-4,4′-DMAR in awake CD-1 male mice.

**Methods:**

We evaluated heart rate, breathing rate, and cardiac (ECG) and respiratory (plethysmography) electrical parameters. The most severe effects were observed after administration of 10 mg/kg (±)*cis*-4,4′-DMAR and coadministration of 1 mg/kg (±)*cis*-4,4′-DMAR and 30 mg/kg (±)*trans*-4,4′-DMAR.

**Results:**

These treatments induced tachycardia and narrow-QRS arrhythmias, characterized by an increase in the corrected QT/QT interval, tachypnea, and a decrease in relaxation time, with varying degrees of intensity, probably due to differences in metabolism. Moreover, immunohistochemical analysis of the heart specimens revealed significant alterations in inflammation/oxidative stress markers in cardiomyocytes and blood vessel walls, accompanied by cytoarchitectural changes.

**Conclusions:**

These findings provide the first evidence of the severe toxicity of 4,4′-DMAR to the cardiac and respiratory systems, highlighting the importance of dosage and stereoisomer coadministration. This serves as a warning of the complex nature and potential dangers of intoxication.

## 1 Introduction

**Figure 1:**
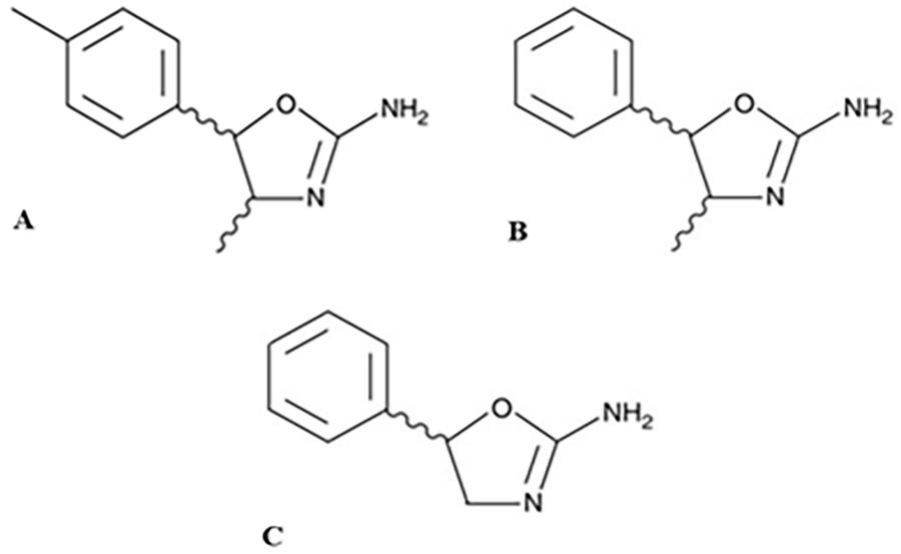
Chemical structures of 4,4′-DMAR (4-methyl-5-(4-methylphenyl)-4,5-dihydrooxazol-2-amine; **A**), 4-MAR (4-methyl-5-phenyl-4,5-dihydrooxazol-2-amine; **B**) and mminorex (5-phenyl-4,5-dihydro-1,3-oxazol-2-amine; **C**) modified (14).

Over the years, the clandestine drug market has steadily expanded, accompanied by an increase in the number of illicit new psychoactive substances (1). Among novel synthetic stimulants, which account for 35% of the total monitored novel psychoactive substances (2), various analogs of the anorexic agent aminorex have gained much popularity because their pharmacological profile is similar to that of amphetamine-type stimulants (3). 4,4′-Dimethylaminorex [4-methyl-5-(4-methylphenyl)-4,5-dihydrooxazol-2-amine] is one of the best-known aminorex analogs (4, 5). It is a substituted synthetic oxazolinic derivative with two chiral centers, yielding four stereoisomers (Figure 1) (6). As a result, it exists as two racemic mixtures: (±)*cis-*4,4′-DMAR and (±)*trans-*4,4′-DMAR, which exhibit different biological characteristics (7, 8). 4,4′-DMAR was first detected in the Netherlands (9). Between 2013 and 2014, 4,4′-DMAR was analytically confirmed in 32 cases of poisoning and death involving adults and adolescents (7, 8, 10), which led to its inclusion under national and international legislation control (4, 5, 9). Nevertheless, it remains widely available on the “Dark Web” in various forms (i.e., white powder or differently colored tablets and pellets) (11, 12). The desired effects of 4,4′-DMAR include feelings of happiness and euphoria, enhanced talkativeness, and increased sociability, similar to those associated with 3,4-methylenedioxymethamphetamine (MDMA), thus indicating that it is a valid alternative despite its stronger serotonin effects (13). However, users have also reported several adverse effects, including hyperthermia, sweating, agitation, convulsion, respiratory impairments, tachycardia, and cardiac arrest (9, 12). Our previous study demonstrated that (*±*)*cis-*4,4′-DMAR dose-dependently induces a series of toxic effects (psychomotor agitation, sweating, salivation, hyperthermia, stimulated aggression, convulsions, and death) in mice. Although (±)*trans*-administration was ineffective, its coadministration with (*±*)*cis-*4,4′-DMAR worsened the above-mentioned effects (14). However, clinical and preclinical investigations of cardiorespiratory toxicity remain limited. On this basis, we have focused on the cardiovascular effects of 4,4′-DMAR, evaluating the possible worsening of toxic effects induced by coadministration of the two racemic forms compared with (*±*)*cis-*4,4′-DMAR alone.

## 2 Methods

### 2.1 Animals

Fifty male outbred ICR (CD-1®) mice (25–30 g, ENVIGO Harlan Italy, Italy) were group-housed (8–10 mice per cage; floor area per animal, 80 cm^2^; minimum enclosure height, 12 cm) under a constant temperature of 20 °C–22 °C and humidity of 45%–55%. Food (Diet 4RF25 GLP; Mucedola, Settimo Milanese, Milan, Italy) and tap water were available *ad libitum* throughout the period the animals spent in their home cages. The daylight cycle was artificially maintained (12:12 h light–dark cycle, lights on between 7 a.m. and 7 p.m.). The exclusive use of male mice was based on scientific literature highlighting a higher incidence of intoxication among men (9). However, further research are needed to investigate potential sex-specific responses to the drug. All experiments were performed during the light phase. The experimental protocols in the present study were performed in accordance with the U.K. Animals (Scientific Procedures) Act 1986, associated guidelines, and the new European Communities Council Directive of September 2010 (2010/63/EU). The experimental protocols were approved by the Italian Ministry of Health (license no. 335/2016-PR) and the Animal Welfare Body of the University of Ferrara. According to the ARRIVE guidelines, efforts were made to minimize the number of animals used, reduce pain and discomfort, and limit the number of experimental subjects. For the cardiorespiratory studies, six mice/group were used for (*±*)*cis-*4,4′-DMAR treatment (saline or three different doses of 1, 3, and 10 mg/kg; total mice used, 24). In the coadministration experiments, six mice/group were used for each treatment (saline, 30 mg/kg (±)*trans-*4,4′-DMAR, and coadministration of 1 mg/kg (*±*)*cis-*4,4′-DMAR and 30 mg/kg (±)*trans-*4,4′-DMAR; total mice used, 18). For electrocardiographic and plethysmographic analyses, four mice/group were used for treatment with 10 mg/kg (*±*)*cis-*4,4′-DMAR and for the coadministration of 1 mg/kg (*±*)*cis-*4,4′-DMAR and 30 mg/kg (±)*trans-*4,4′-DMAR (total mice used, 8).

### 2.2 Drug preparation and dose selection

*Cis* and *trans* 4,4′-DMAR were obtained from the synthesis of 4-methylpropiophenone prior to their regulation under existing legislation (14). In particular, the two isomers, (±)*cis*-4,4′-DMAR and (±)*trans-*4,4′-DMAR, were synthesized as previously described by Brandt and coworkers (8). Drugs were initially dissolved in absolute ethanol (final concentration, 2%) and Tween 80 (2%) and then brought to a final volume with saline (0.9% NaCl). A solution containing ethanol, Tween 80, and saline was used as the vehicle. The selected doses of (*±*)*cis-*4,4′-DMAR (1, 10, and 30 mg/kg), (±)*trans-*4,4′-DMAR (30 mg/kg), and the coadministration regimen (1 mg/kg (*±*)*cis-*4,4′-DMAR+30 mg/kg (±)*trans-*4,4′-DMAR) were based on the analysis of the effect of single enantiomers previously performed by Tirri and colleagues (14). The drugs were administered as follows: intraperitoneal injection (i.p.) at a volume of 4 µL/g body weight.

### 2.3 Evaluation of cardiorespiratory changes in mice

To monitor cardiorespiratory parameters in awake, freely moving animals without using invasive instruments and handling, a sensor-equipped collar was used to measure heart rate (HR), breathing rate (BR), oxygen saturation (SO_2_), and pulse distension at an acquisition frequency of 15 Hz (15–17). During the experiment, mice were allowed to freely move within their cages (30 cm × 30 cm × 20 cm) without access to food or water while being monitored by the sensor collar using MouseOx Plus software (STARR Life Sciences® Corp, Oakmont, PA). During the first hour of acclimation, a fake collar identical in design to the experimental collar but lacking a sensor was used to minimize potential stress on the mouse during the experiment. The sensor-equipped collar was then applied, and baseline parameters were monitored for 60 min. Subsequently, the drugs or vehicle were administered, and the data were recorded for 5 h.

Electrocardiographic (ECG) and plethysmographic parameters were collected from conscious animals using a non-invasive telemetry system for the acquisition of ECG and respiration data in rodents (ecgTUNNEL for rodents, Emka Technologies, Paris, France), which eliminates the need for surgery and anesthesia, at an acquisition frequency of 1,000 Hz. All ECG recording sessions were performed during the daytime, and the data were analyzed using iox2 data acquisition analysis software (Emka Technologies, Paris, France). Each animal was placed inside the tunnel, which was then closed to ensure proper restraint. To minimize stress-related effects, the animals were allowed to remain in the restraint system for 1 min before recording. Indeed, direct observation of the animals, along ECG and plethysmography traces, confirmed that they were calm and that their heart rate and breathing rate were stable. The experiment provided 15 min of baseline recording, followed by 45 min of recording during vehicle or drug exposure. For data acquisition, repeated measurements were performed on the same animal at each time point, and data for the same animal were collected over different weekly intervals.

### 2.4 ECG and plethysmographic data evaluation

For ECG and plethysmography, emka ECG and Plethysmography Analyzer software were used to analyze tracings recorded during data acquisition. ECG measurements were recorded in two experimental phases (before and after treatment based, on the HR and BR values from MouseOx). Based on the MouseOx profile, a 1-min time window was selected during emka analysis to evaluate the recorded parameters at an acquisition frequency of 1,000 Hz. For the ECG analysis, the following parameters were included: HR (bpm; heart rate), RR interval (ms; time between two consecutive R-wave peaks), PR interval (ms; time from the onset of the P wave to the start of the QRS complex), QRS (ms; time from the start to the end of the QRS complex), and QT interval (ms; time from the beginning of the QRS complex to the end of the T wave). To better evaluate the duration of the ventricular electrocardiogram (QT interval) without the influence of HR, the corrected QT (QTcF) was calculated using the Fridericia formula (QTcF = QT/∛RR) (15). ECG recordings were exported for further analysis.

For plethysmography, FR_IE (bpm; breathing frequency), BB_IE [ms; breath length (BB) computed by adding inspiration and expiration duration], TV (mL/s; tidal inspiration volume), EXP/INSP_V_RATIO AVER (ratio of expired volume to tidal volume), and RT (ms; relaxation time) were determined. All parameters were analyzed using iox2 analysis software. ECG data were analyzed in relation to the HR dose–response curve and showed electrical variations, referred to as HR variations, in vehicle- and drug-treated mice. Plethysmography data were analyzed in relation to BR dose–response curves and showed electrical variations, referred to BR variations, in vehicle-treated mice and mice treated with 10 mg/kg (±)*cis*-4,4′-DMAR and the coadministration regimen [1 mg/kg (±)*cis*-4,4′-DMAR  + 30 mg/kg (±)*trans*-4,4′-DMAR].

### 2.5 Cardiac tissue sampling: histopathology and immunohistochemistry

#### 2.5.1 Specimen preparation

After the mice were sacrificed by cervical dislocation, their hearts were quickly excised as previously reported (18), rinsed in 0.9% NaCl, and fixed by immersion overnight at room temperature in 10% neutral buffered formalin. The tissues were then kept in absolute ethanol, followed by acetone, and finally embedded in Bio Plast Plus (Bio-Optica, Milan, Italy). Using a manual rotatory microtome, 5-μm-thick sagittal heart sections were prepared, mounted on polysine-coated slides, and processed for histological and immunohistochemical analyses.

#### 2.5.2 Morphological and histopathological examination

To reveal cardiac tissue cytoarchitecture and assess potential structural alterations by light microscopy, hematoxylin and eosin (H&E) and Picrosirius Red (PSR) staining were performed as previously described (19, 20). Briefly, tissue sections were processed as follows: (i) H&E staining: sections were stained with Carazzi's hematoxylin (Bio-Optica Milano S.p.A., Milano, Italy) for 10 min, washed in running tap water for 20 min, and counterstained with 1% eosin solution (Bio-Optica Milano S.p.A., Milano, Italy); nucleic acids stained violet to dark blue, while the proteins stained pink to red; (ii) PSR staining: sections were stained with Picrosirius Red solution (0.1% Sirius Red in saturated aqueous picric acid) for 1 h, followed by washing in 5% acidified water. Finally, the stained sections were dehydrated in ethanol, cleared in xylene, and mounted using Eukitt (Kindler, Freiburg, Germany).

#### 2.5.3 Light microscopy and immunohistochemistry

To prevent possible staining discrepancies caused by minor procedural changes, immunocytochemical reactions were conducted simultaneously on slides from the different experimental groups. Immunohistochemistry was performed using commercial antibodies on murine heart specimens to identify and localize the presence and distribution of the following markers: (i) interleukin-6 (IL-6), (ii) nuclear factor erythroid 2-related factor 2 (Nrf2), and (iii) 70-kDa heat shock protein (Hsp70).

After being deparaffinized in xylene and rehydrated, cardiac tissue sections of vehicle-, (±)*cis*-4,4′-DMAR (10 mg/kg)-, (±)*cis*-4,4′-DMAR (1 mg/kg)-, and (±)*trans*-4,4′-DMAR (30 mg/kg)-treated mice were subjected to antigen retrieval and then incubated overnight at room temperature in a dark moist chamber with selected monoclonal and polyclonal primary antibodies (Table 1) diluted in PBS. Subsequently, appropriate biotinylated secondary antibodies (Table 1) and an avidin–biotin horseradish peroxidase complex (Vector Laboratories, Burlingame, CA, USA) were used to detect antigen/antibody interaction sites. The 3,3′-diaminobenzidine tetrahydrochloride peroxidase substrate (Sigma, St. Louis, MO, USA) was used as the chromogen, while nuclear counterstaining was performed using Carazzi's hematoxylin.

**Table 1:** Primary/secondary antibodies employed for light microscopy experimental procedures.

	Antigen	Immunogen	Manufacturer, species, mono/polyclonal, Cat./Lot. no., RRID	Dilution
Primary antibodies	Anti-interleukin-6 (M-19)	Purified antibody raised against a peptide mapping at the C-terminus of murine IL-6	Santa Cruz Biotechnology (Santa Cruz, CA, USA), goat polyclonal IgG, Cat# sc-1265, RRID: AB_2127470	1:100
	Anti-nuclear factor erythroid 2-related factor 2	Purified antibody raised against a peptide within human Nrf2 aa 550 to the C-terminus	Abcam (Cambridge, UK), rabbit polyclonal IgG, Cat#ab31163, RRID: AB_881705	1:100
	Anti-HSP70/HSC 70 (5A5)	Purified antibody raised against recombinant HSP 70/HSC 70 of human origin	Santa Cruz Biotechnology (Santa Cruz, CA, USA), mouse monoclonal IgG, Cat# sc-32239, RRID: AB_627759	1:100
Secondary antibodies	Biotinylated goat anti-rabbit IgG	Gamma immunoglobulin	Vector Laboratories (Burlingame, CA, USA), goat, lot# PK-6101, RRID: AB_2336820	1:200
	Biotinylated rabbit anti-goat IgG	Gamma immunoglobulin	Vector Laboratories (Burlingame, CA, USA), rabbit, Cat# PK-6105, RRID: AB_2336824	1:200
	Biotinylated horse anti-mouse IgG	Gamma immunoglobulin	Vector Laboratories (Burlingame, CA, USA), horse, Cat# PK-6102, RRID: AB_2336821	1:200

The sections were then dehydrated in ethanol, cleared in xylene, and mounted in Eukitt (Kindler, Freiburg, Germany). For negative controls, some tissues were incubated with PBS in the absence of primary antibodies; no immunoreactivity was detected under these conditions.

#### 2.5.4 Microscopes, imaging systems, and histochemical/immunohistochemical measurements

After morphological, histochemical, and immunohistochemical procedures, the slides were observed and scored using a bright-field Optika B-1000 (Optika S.r.l., BG, Italy). Five slides (approximately 18–20 randomized sections) per animal were analyzed, and five microscopic fields were examined in each section for each mouse per condition. Images were recorded using a C-P20CC digital camera (Optika S.r.l., BG, Italy).

Cardiac tissue specimens from all experimental groups displayed varying degrees of immunolabeling, and the figures depict the most representative changes for each immunohistochemical reaction. Labeling intensity, expressed as optical density (OD), was assessed in three randomized images/sections (making at least 10 measurements/image) per five slides per animal in each experimental group, with the operator blinded to the experimental conditions. The analysis was performed using ImageJ software (ImageJ 1.46p; NIH, Bethesda, MA, USA), and the mask shape was adjusted depending on the spatial distribution of the cell type and/or tissue specimen being analyzed; labeling intensity was measured as the mean intensity value over the area. The results were recorded in Microsoft Office Excel Software spreadsheets.

For the morphological and histochemical evaluations, the following additional measurements were performed: arteriole wall thickness (µm) and vessel-labeled area (wall area in µm^2^/labeled area in µm^2^).

### 2.6 Statistical analysis

Data related to heart rate [heartbeat per min (bpm)] and breathing rate [respiratory rate per minute (rpm)] were expressed as % changes in basal values. The effects of different concentrations of each substance over time were analyzed using two-way ANOVA followed by Bonferroni's test for multiple comparisons, as appropriate. Cardiovascular data are expressed as the percentage of baseline with the mean ± SEM of six independent experiments. ECG and plethysmography recordings were analyzed using Student's *t*-test for each basal and after-treatment parameter comparison, and data are expressed as mean overall effects of 15 evaluations for baseline and after treatment, with mean ± SEM of 15 different evaluations (evaluations were obtained based on 1-min windows).

Histological, histochemical, and immunohistochemical data are expressed as mean ± SEM. The Shapiro–Wilk and Kolmogorov–Smirnov tests were used to assess and confirm the normality of the parameters. The data were then analyzed to determine whether differences were statistically significant. For normally distributed data, analysis was conducted using one-way ANOVA followed by Bonferroni's *post-hoc* test for multiple comparisons. For non-normally distributed results, analysis was performed using the Kruskal–Wallis test followed by Dunn's test. For all statistical analyses, performed using GraphPad Prism 8.0 (GraphPad Software, Inc., CA, USA), differences were considered statistically significant at *p* < 0.05 (*), *p* < 0.01 (**), and *p* < 0.001 (***).

## 3 Results

### 3.1 Cardiac parameters of (±)*cis*-4,4′-DMAR

**Figure 2:**
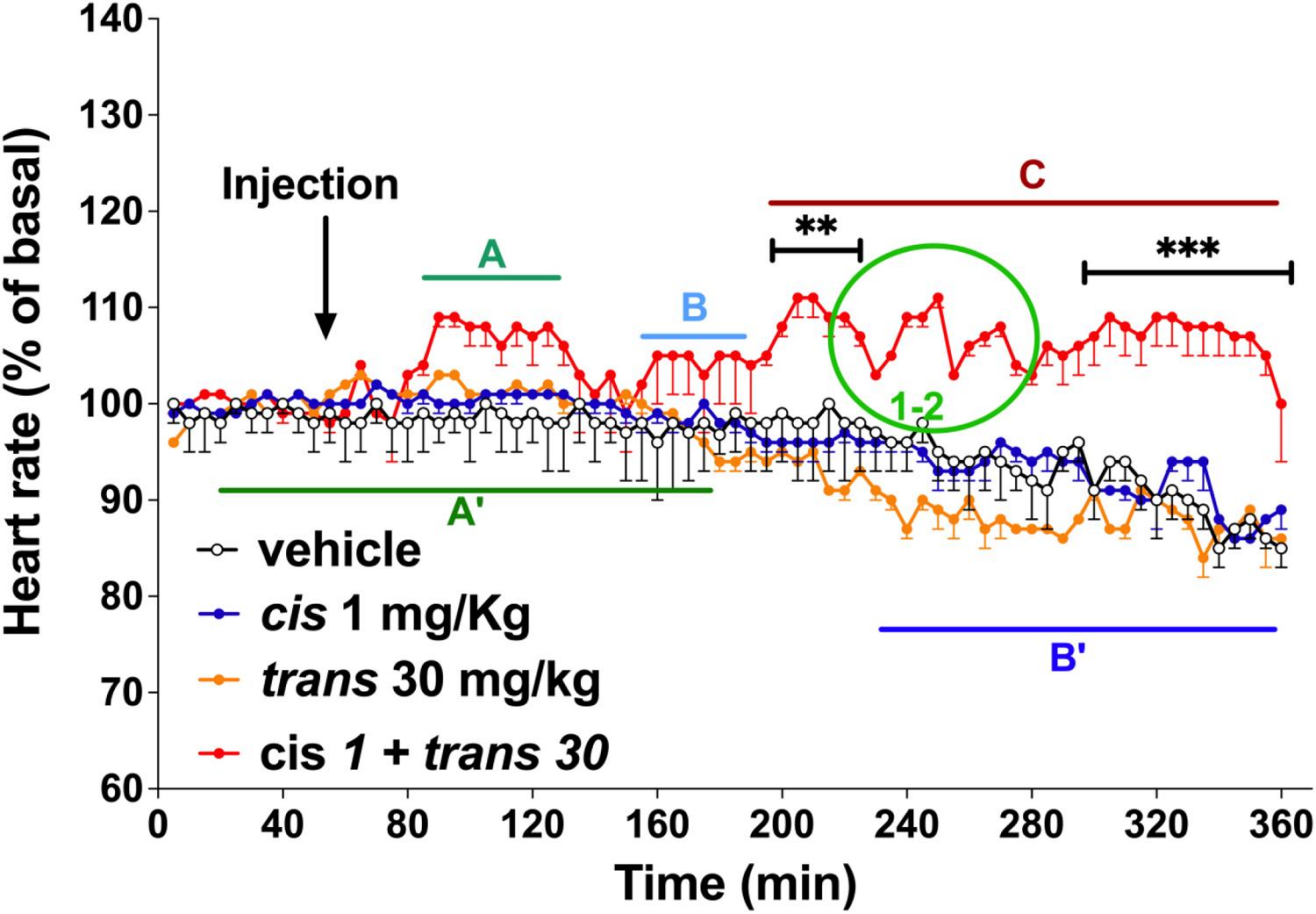
Effect of systemic administration of (±)*cis*-4,4′-DMAR (1, 10, and 30 mg/kg) on heart rate. Data are expressed as a percentage of baseline (mean ± SEM; *n* = 6 per group). Statistical analysis was performed using two-way ANOVA followed by Bonferroni's test for multiple comparisons (**p < 0.01, ***p < 0.001). Vehicle and 10 mg/kg treatment dose–response curves are marked by letters (A′ and B′ for the vehicle and A, B, C, and 1–2 for 10 mg/kg treatment), to evaluate effects on ECG electrical parameters and tracings. Capital letters indicate significant heart rate variations. Numbers highlight potential arrhythmic events at the specific time points marked by green circles.

**Figure 3:**
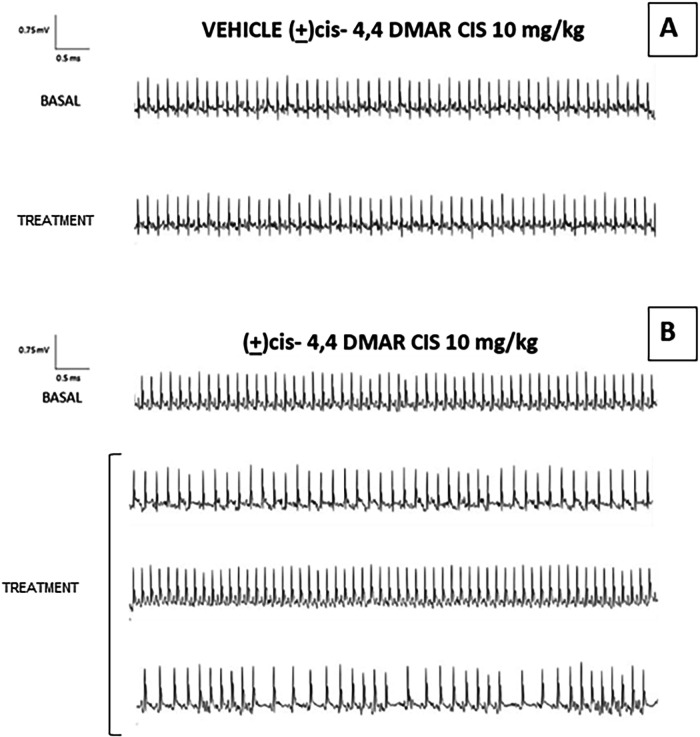
ECG tracings of vehicle-treated mice (**A**), mice treated with (±)*cis*-4,4′-DMAR (10 mg/kg; **B**), and representative arrhythmias induced by (±)*cis*-4,4′-DMAR (10 mg/kg). Tracings correspond to the heart-rate dose–response curve shown in Figure 2. Recordings were obtained using the ecgTUNNEL system (emka Technologies, Paris, France), and tracings were exported after analysis with iox2 software.

Systemic administration of (*±*)*cis-*4,4′-DMAR significantly affected cardiac parameters in mice, particularly at the highest dose (Figure 2); a significant effect of treatment (*F*_3, 1,440_ = 361.5, *p* < 0.0001), time (*F*_71, 1,440_ = 5.108, *p* < 0.0001), and time × treatment interaction (*F*_213, 1,440_ = 6.035, *p* < 0.0001) was observed. The HR of vehicle-treated mice (715.44 ± 2.51 bpm) physiologically decreased during the 3-h observation period (similar to naïve mice; data not shown). Systemic administration of (*±*)*cis-*4,4′-DMAR increased the HR by ∼10% 1-h after injection, followed by a further increase of ∼30% 3 h after injection only at the highest dose tested (10 mg/kg). Subsequently, the HR returned to ∼20% during the last 2 h. The vehicle and (*±*)*cis-*4,4′-DMAR (10 mg/kg) dose–response curves are marked by letters (A′ and B′ for vehicle and A, B, and C for (*±*)*cis-*4,4′-DMAR). These letters correspond to the ECG tracings (Figure 3), illustrating the main heart patterns of the vehicle (Figure 3A), the initial slight bradycardia, and the marked tachycardia induced by the administration of (*±*)*cis-*4,4′-DMAR (Figure 3B). Moreover, arrhythmias induced by (*±*)*cis-*4,4′-DMAR were also observed (Figure 3C). Finally, the ECG electrical parameters relative to the dose–response curve (Figure 2) for vehicle-treated mice and mice receiving (*±*)*cis-*4,4′-DMAR were reported (Table 2). Baseline cardiac electrical parameters showed a slight decrease in HR (Table 2; significant effect of treatment, *t* = 7.373, df = 28, *p* < 0.0001) with a consequent increase in the RR interval (Table 2; significant effect of treatment, *t* = 6.767, df = 28, *p* < 0.0001); however, these values did not differ from untreated mice ECG parameters (data not shown). After 10 mg/kg (*±*)*cis-*4,4′-DMAR administration, three different HR variations were identified along the dose–response curve (Figure 2). Immediately after the injection (Table 2), the HR decreased by ∼10% (significant effect of treatment, *t* = 11.30, df = 28, *p* < 0.0001) and the RR interval increased by ∼10% (significant effect of treatment, *t* = 11.45, df = 28, *p* = 0.0001), while other parameters did not change compared to the baseline values. Relative to the dose–response curve, the points marked with letter B (Table 2) showed a mild HR increase of ∼5% (significant effect of treatment, *t* = 7.629, df = 28, *p* < 0.0001), an RR interval decrease of ∼5% (significant effect of treatment, *t* = 7.373, df = 28, *p* < 0.0001), and a prolonged QTcF interval of ∼12% (significant effect of treatment, *t* = 2.414, df = 28, *p* = 0.0226). Electrical parameters referring to an increase in HR marked with letter C (Table 2) indicated an increase in HR of ∼11% (significant effect of treatment, *t* = 14.48, df = 28, *p* < 0.0001), RR interval decrease of ∼8% (significant effect of treatment, *t* = 11.21, df = 28, *p* < 0.0001), characterized by a prolonged QT interval of ∼18% (significant effect of treatment, *t* = 3.801, df = 28, *p* = 0.0007), and a prolonged QTcF interval of ∼27% (significant effect of treatment, *t* = 5.015, df = 28, *p* < 0.0001).

**Table 2:** Effect of systemic administration of vehicle (A′ and B′) and (*±*)*cis-*4,4′-DMAR 10 mg/kg (A, B, and C) on cardiac electrical parameters (ECG analysis).

ECG parameters						
Vehicle						
	HR	RR interval	PR interval	QRS duration	QT interval	QTcF interval
BASAL	723.12 ± 1.99	83.2 ± 0.25	22.47 ± 2.71	9.55 ± 0.26	27.02 ± 1.27	61.13 ± 3.71
A′	721.77 ± 1.91	83.32 ± 0.22	21.58 ± 2.76	9.23 ± 0.24	27.07 ± 1.36	61.77 ± 3.42
B′	692.01 ± 3.73***	87.08 ± 0.52***	22.28 ± 2.94	10.05 ± 0.26	29.32 ± 1.46	64.76 ± 3.35
(±)*cis*-4,4′-DMAR (10 mg/kg)						
BASAL	711.34 ± 3.88	84.40 ± 0.46	22.91 ± 0.62	9.95 ± 0.15	28.77 ± 0.58	63.13 ± 1.43
A	646.10 ± 4.27***	93.06 ± 0.60***	24.41 ± 2.51	9.80 ± 0.25	30.86 ± 1.28	68.15 ± 2.18
B	746.41 ± 2.46***	80.48 ± 0.27***	21.23 ± 2.78	10.09 ± 0.39	30.89 ± 1.37	71.51 ± 3.16
C	782.04 ± 2.96***	78.19 ± 0.31***	25.19 ± 2.23	10.05 ± 0.25	34.34 ± 1.34**	80.38 ± 3.13***

Data are relative to the (*±*)*cis-*4,4′-DMAR (10 mg/kg) HR dose–response curve (Figure 2). Data are expressed as mean overall effects across 15 evaluations for baseline and after vehicle or treatment in four mice. Data are analyzed using iox2 software and expressed as mean ± SEM of evaluations recorded in a 1-min time window, capturing data at an acquisition frequency of 1,000 Hz. Statistical analysis was performed by Student's *t*-test for each basal and after-treatment parameter comparisons. HR (bpm; heart rate), RR interval (ms; time between two consecutive peaks), PR (ms; time from the onset of the P wave to the start of the QRS complex), QRS (ms; is the time from the start to the end of the QRS complex), QT interval (ms; time from the beginning of the QRS complex to the end of the T wave), QTcF interval (QT/∛RR).

** *p* < 0.01.

*** *p* < 0.001.

### 3.2 Cardiac parameters of coadministration (1 mg/kg (±)*cis*-4,4′-DMAR +30 mg/kg (±)*trans*-4,4′-DMAR)

**Figure 4:**
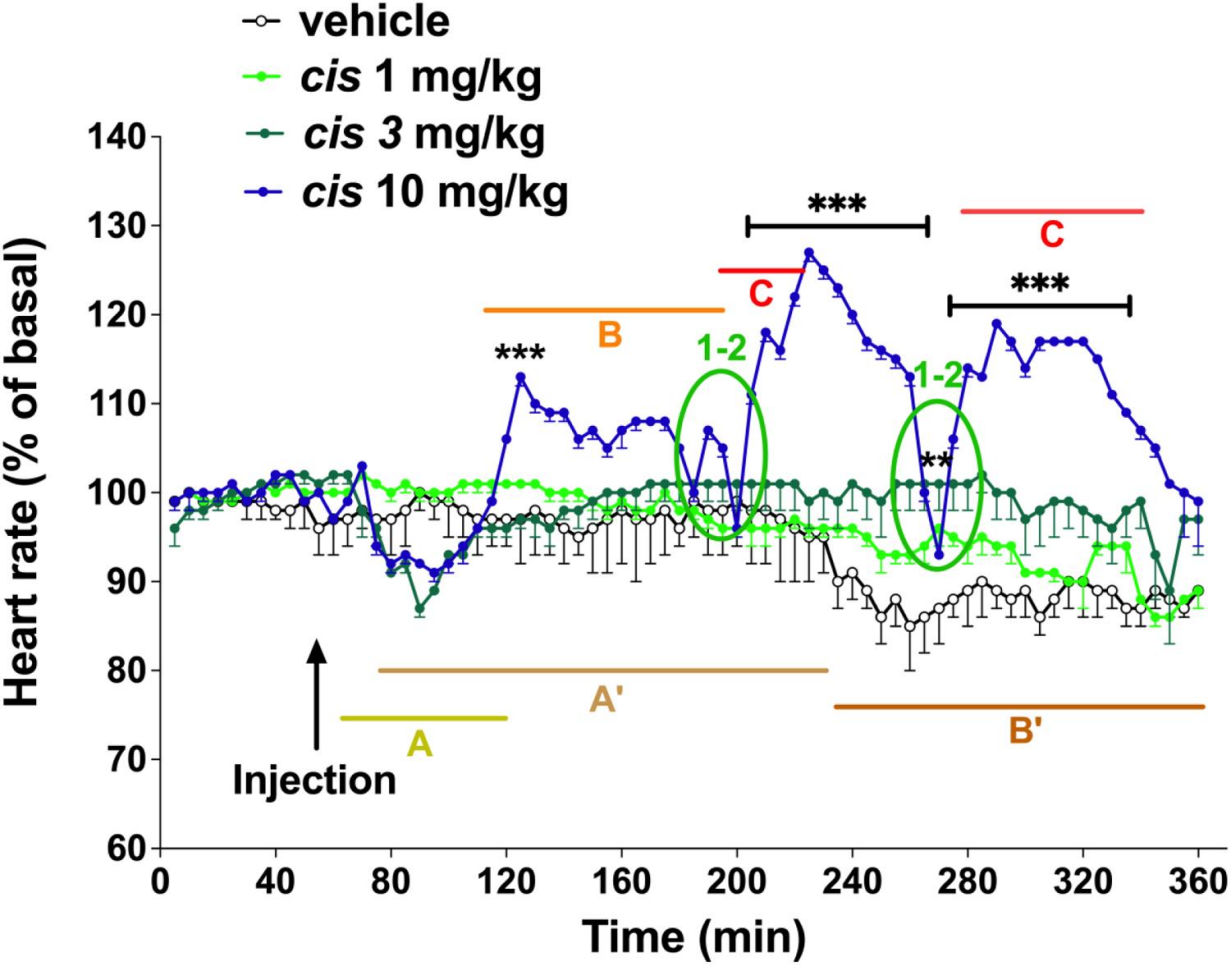
Effect of systemic administration of (±)*cis*-4,4′-DMAR (1 mg/kg), (±)*trans*-4,4′-DMAR (30 mg/kg), and their coadministration on heart rate. Data are expressed as a percentage of baseline (mean ± SEM; *n* = 6 per group). Statistical analysis was performed using two-way ANOVA followed by Bonferroni's test for multiple comparisons (**p < 0.01, ***p < 0.001). Vehicle and coadministration treatment dose–response curves are marked by letters (A′ and B′ for the vehicle; A, B, and 1–2 for the coadministration treatment) to identify the time points corresponding to ECG electrical parameters and tracings. Capital letters indicate significant heart rate variations. Numbers highlight potential arrhythmic events at the specific time points marked by green circles.

**Figure 5:**
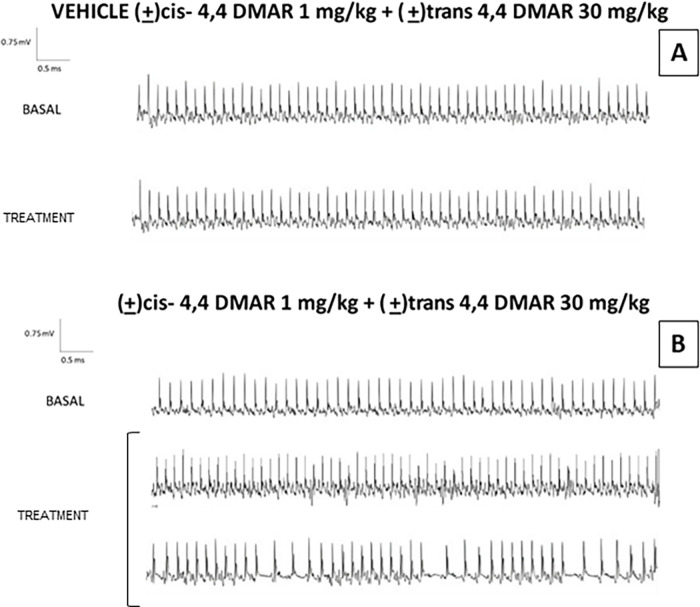
ECG tracings of vehicle-treated mice (**A**), mice receiving the coadministration of (±)*cis*-4,4′-DMAR (1 mg/kg) and (±)*trans*-4,4′-DMAR (30 mg/kg; **B**), and representative supraventricular arrhythmia (1) and atrial fibrillation (2) induced by coadministration. Tracings correspond to the heart-rate dose–response curve shown in Figure 4. Recordings were obtained using the ecgTUNNEL system (emka Technologies), and tracings were exported after analysis with iox2 software.

Systemic coadministration of (*±*)*cis-*4,4′-DMAR (1 mg/kg) and (±)*trans-*4,4′-DMAR (30 mg/kg) significantly affected cardiac parameters in mice (Figure 4); a significant effect of treatment (*F*_3, 1,440_ = 261.3, *p* < 0.0001), time (*F*_71, 1,440_ = 6.155, *p* < 0.0001), and time × treatment interaction (*F*_213, 1,440_ = 2.497, *p* < 0.0001) was observed. The HR of vehicle-treated mice (651.23 ± 1.73 bpm) decreased during the last 3-h observation, similar to the physiological trend onserved in untreated mice (data not shown). Administration of (*±*)*cis-*4,4′-MAR (1 mg/kg) and (±)*trans-*4,4′-DMAR (30 mg/kg) alone did not produce significant changes compared with vehicle-treated animals. However, coadministration of 1 mg/kg (*±*)*cis-*4,4′-DMAR and 30 mg/kg (±)*trans-*4,4′-DMAR increased the HR by ∼10% for up to 120 min, which mildly decreased up to 200 min and then increased by ∼20% compared to the baseline during the last 3 h of the experiment. The vehicle and the coadministration dose–response curve were marked by the letters A′ and B′ for the vehicle and A, B, and C for coadministration treatment. These letters correspond to the ECG tracings (Figure 5) and represent the main HR trends of the vehicle (Figure 5A), the tachycardic effect (Figure 5B), and narrow-QRS tachyarrhythmias, including paroxysmal supraventricular tachycardia and atrial fibrillation (Figure 5C), induced by coadministration. Finally, the ECG electrical parameters relative to the dose–response curve (Figure 4) for the vehicle-treated mice and mice receiving the coadministration of (*±*)*cis-*4,4′-DMAR (1 mg/kg) and (±)*trans-*4,4′-DMAR (30 mg/kg) were reported (Table 3). Vehicle-treated mice showed a decrease in HR (Table 3; significant effect of treatment, *t* = 8.025, df = 28, *p* < 0.0001) and an increase in the RR interval (Table 3; significant effect of treatment, *t* = 7.366, df = 28, *p* < 0.0001); however, these values did not differ from untreated mice ECG parameters (data not shown). After coadministration (1 mg/kg (*±*)*cis-*4,4′-DMAR +30 mg/kg (±)*trans-*4,4′-DMAR), the HR increased, while the RR interval decreased by ∼10% (Table 3; HR: significant effect of treatment, *t* = 7.763, df = 28, *p* < 0.0001; RR: significant effect of treatment, *t* = 6.940, df = 28, *p* < 0.0001), ∼5% (Table 3; HR: significant effect of treatment, *t* = 3.801, df = 28, *p* = 0.0007; RR: significant effect of treatment, *t* = 3.612, df = 28, *p* = 0.0012), and ∼12% (Table 3; HR: significant effect of treatment, *t* = 9.873, df = 28, *p* < 0.0001; RR: significant effect of treatment, *t* = 8.571, df = 28, *p* < 0.0001), at time points marked with A, B, and C on the dose–response curve, respectively. At points A and C, QT and QTcF also showed prolongation. In particular, at point A, QT interval prolonged by ∼10% (Table 3; significant effect of treatment, *t* = 2.339, df = 28, *p* = 0.0267) and QTcF prolonged by ∼13% (Table 3; significant effect of treatment, *t* = 3.452, df = 28, *p* = 0.0018), while at point C, QT prolonged by ∼11% (Table 3; significant effect of treatment, *t* = 2.078, df = 28, *p* = 0.0470) and QTcF prolonged by ∼16% Table 3; significant effect of treatment, *t* = 2.736, df = 28, *p* = 0.0107).

**Table 3:** Effect of systemic administration of vehicle (A′ and B′) and coadministration of (±)*cis*-4,4′-DMAR (1 mg/kg) and (±)*trans*-4,4′-DMAR (30 mg/kg) (A, B, and 1–2) on cardiac electrical parameters (ECG analysis).

ECG parameters						
Vehicle						
	HR	RR interval	PR interval	QRS duration	QT interval	QTcF interval
BASAL	644.29 ± 3.34	93.45 ± 0.51	19.03 ± 3.27	10.01 ± 0.03	26.65 ± 1.02	47.37 ± 1.79
A′	643.20 ± 5.12	95.06 ± 0.44	22.05 ± 3.72	9.98 ± 0.32	25.95 ± 1.15	48.32 ± 2.08
B′	594.03 ± 5.29***	102.00 ± 1.04***	22.73 ± 2.92	9.95 ± 0.30	25.66 ± 1.10	55.06 ± 2.41
(±)*cis*-4,4′-DMAR (1 mg/kg) and (±)*trans*-4,4′-DMAR (30 mg/kg)						
BASAL	661.48 ± 7.97	91.03 ± 1.72	19.33 ± 0.82	10.03 ± 0.07	25.76 ± 0.23	57.33 ± 0.60
A	726.86 ± 2.70***	82.59 ± 0.32***	23.84 ± 2.97	9.45 ± 0.31	28.29 ± 1.05*	64.96 ± 3.12**
B	692.25 ± 1.38**	86.75 ± 0.18**	21.96 ± 3.32	10.21 ± 0.31	27.03 ± 1.03	61.09 ± 2.80
C	743.00 ± 2.13***	80.79 ± 0.23***	23.74 ± 2.66	10.33 ± 0.32	28.73 ± 1.41*	66.49 ± 3.29*

Data are relative to the coadministration of (±)*cis*-4,4′-DMAR 1 mg/kg and (±)*trans*-4,4′-DMAR 30 mg/kg HR dose–response curve (Figure 4). Data are expressed as the mean overall effects across 15 evaluations for baseline and after vehicle or JWH-018 treatment in four mice. Data are analyzed using iox2 software and expressed as mean ± SEM of evaluations recorded in a 1-min time window, capturing data at an acquisition frequency of 1,000 Hz. Statistical analysis was performed using Student's *t*-test for each basal and after-treatment parameter comparisons. HR (bpm; heart rate), RR interval (ms; time between two consecutive peaks), PR interval (ms; time from the onset of the P wave to the start of the QRS complex), QRS (ms; is the time from the start to the end of the QRS complex), QT interval (ms; time from the beginning of the QRS complex to the end of the T wave), QTcF interval (QT/∛RR).

* *p* < 0.05.

** *p* < 0.01.

*** *p* < 0.001.

### 3.3 Respiratory parameters of (±)*cis*-4,4′-DMAR

**Figure 6:**
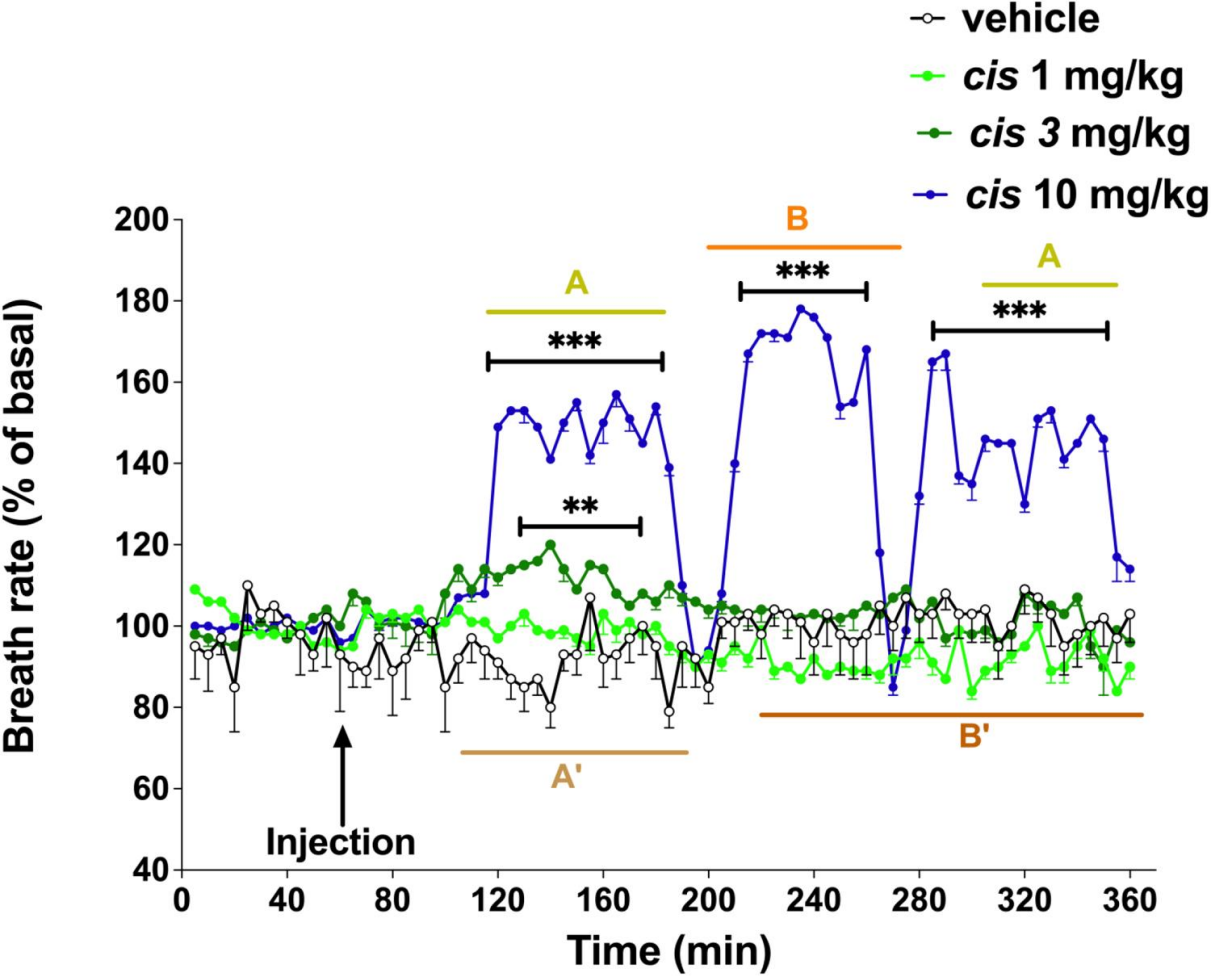
Effect of systemic administration of (±)*cis*-4,4′-DMAR (1, 10, and 30 mg/kg) on breathing rate. Data are expressed as a percentage of baseline, with mean ± SEM from six different evaluations for each group. Statistical analysis was performed by two-way ANOVA followed by Bonferroni's test for multiple comparisons (**p < 0.01, ***p < 0.001). Vehicle and 10 mg/kg treatment dose-response curves are marked by letters (A′ and B′ for the vehicle and A and B for 10 mg/kg treatment) to evaluate effects with plethysmography electrical parameters.

Systemic administration of (*±*)*cis-*4,4′-DMAR (1, 3, and 10 mg/kg) affected the breathing rate in the mice (Figure 6). Baseline breathing rate activity (257 ± 14 brpm) did not change in vehicle-treated animals over the 6-h observation, and the effects were similar to those observed in naïve untreated animals (data not shown). The baseline breathing rate increased after (*±*)*cis-*4,4′-DMAR administration (Figure 6); a significant effect of treatment (*F*_3, 1,440_ = 1,083, *p* < 0.0001), time (*F*_71, 1,440_ = 11.98, *p* < 0.0001), and time × treatment interaction (*F*_213, 1,440_ = 12.99, *p* < 0.0001) was observed. In particular, at a dose of 3 mg/kg, the breathing rate increased by ∼10% 1 h after injection, whereas the highest dose (10 mg/kg) increased the breathing rate by ∼50% at 1 h, ∼60% at 2 h, and ∼40% during the last hours of the experiment. The dose–response curve sections, which were analyzed by the emka plethysmography system, were labeled with the letters A′ and B′ for the vehicle and A and B for (*±*)*cis-*4,4′-DMAR. Respiratory parameters were evaluated after vehicle administration and (*±*)*cis-*4,4′-DMAR (Table 4).

**Table 4:** Effect of systemic administration of vehicle (A′ and B′) and (±)*cis*-4,4′-DMAR (10 mg/kg) (A and B) on respiratory electrical parameters (plethysmography analysis).

Plethysmography parameters					
Vehicle					
	fr_ie	BB_ie	TV	exp/insp_V_ratio_aver	RT
BASAL	256.70 ± 5.30	239.03 ± 4.93	0.11 ± 0.0023	−0.46 ± 0.55	71.93 ± 4.60
A′	248.35 ± 4.62	246.27 ± 4.86	0.11 ± 0.002	−0.04 ± 0.46	81.87 ± 2.82
B′	258.04 ± 12.89	244.07 ± 11.09	0.12 ± 0.08	−0.46 ± 0.26	78.33 ± 5.40
(±)*cis*-4,4′-DMAR (10 mg/kg)					
BASAL	248.99 ± 6.43	250.71 ± 6.25	0.14 ± 0.007	−0.12 ± 0.7	98.14 ± 4.52
A	387.12 ± 10.41***	160.54 ± 4.51***	0.13 ± 0.004	−0.39 ± 3.57	67.61 ± 3.48***
B	426.16 ± 16.86***	146.82 ± 6.14***	0.14 ± 0.007	0.58 ± 5.34	59.98 ± 4.44***

Data are relative to the (±)*cis*-4,4′-DMAR BR dose–response curve (Figure 6). Data are expressed as the mean overall effects across 15 evaluations for baseline and after vehicle or JWH-018 treatment in four mice. Data are analyzed using iox2 software and expressed as mean ± SEM of evaluations recorded in a 1-minute time window, capturing data at an acquisition frequency of 1,000 Hz. Statistical analysis was performed using Student's *t*-test for each basal and after-treatment parameter comparisons. FR_IE (bpm; breathing frequency); BB_IE (ms; breath length computed by adding inspiration and expiration duration); TV (mL/s; tidal inspiration volume); EXP/INSP_V_RATIO AVER (ratio of expired volume/tidal volume); RT (ms; relaxation time).

*** *p* < 0.001.

Respiratory electrical parameters did not change in basal, vehicle-, and postvehicle-treated mice compared with normal plethysmography parameters observed in mice (data not shown). After (*±*)*cis-*4,4′-DMAR administration, at each point marked, breathing frequency (fr_ie) increased by ∼55% (Table 4; significant effect of treatment, *t* = 11.28, df = 28, *p* < 0.0001) and ∼71% (Table 4; significant effect of treatment, *t* = 9.817, df = 28, *p* < 0.0001), BB decreased by ∼36% (Table 4; significant effect of treatment, *t* = 11.70, df = 28, *p* < 0.0001) and ∼42% (Table 4; significant effect of treatment, *t* = 11.86, df = 28, *p* < 0.0001), and RT decreased by ∼33% (Table 4; significant effect of treatment, *t* = 5.349, df = 28, *p* < 0.0001) and ∼39% (Table 4; significant effect of treatment, *t* = 6.015, df = 28, *p* < 0.0001).

### 3.4 Respiratory parameters of coadministration (1 mg/kg (±)*cis*-4,4′-DMAR +30 mg/kg (±)*trans*-4,4′-DMAR)

**Figure 7:**
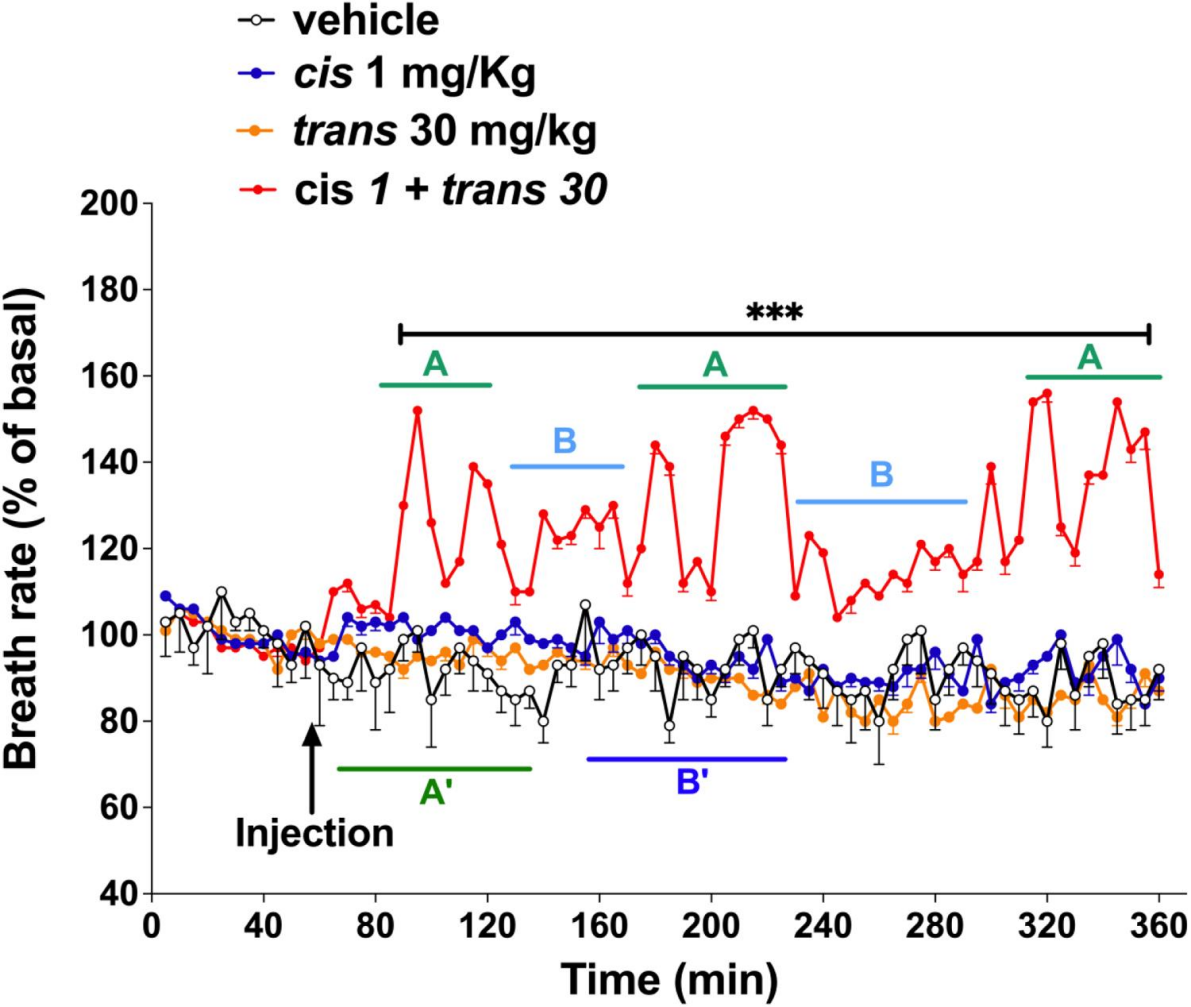
Effect of systemic administration of (±)*cis*-4,4′-DMAR (1 mg/kg), (±)*trans*-4,4′-DMAR (30 mg/kg), and their coadministration on breathing rate. Data are expressed as a percentage of baseline, with mean ± SEM from six different evaluations for each group. Statistical analysis was performed by two-way ANOVA followed by Bonferroni's test for multiple comparisons (***p < 0.001). Vehicle and 10 mg/kg treatment dose–response curves are marked by letters (A′ and B′ for vehicle and A and B for treatment) to evaluate effects with plethysmography electrical parameters.

Systemic coadministration of (*±*)*cis-*4,4′-DMAR (1 mg/kg) and (±)*trans-*4,4′-DMAR (30 mg/kg) affected the breathing rate in mice (Figure 7). Baseline breathing rate slightly decreased only during the initial hours of the experiment and remained similar to that observed in naïve untreated mice (data not shown). Administration of (*±*)*cis-*4,4′-DMAR (1 mg/kg) and (±)*trans-*4,4′-DMAR (30 mg/kg) alone did not change the breathing rate compared with vehicle, but their coadministration increased the breathing rate by ∼50% at 30 min, 2 h, and 4 h after injection (Figure 7); a significant effect of treatment (*F*_3, 1,440_ = 849.7, *p* < 0.0001), time (*F*_71, 1,440_ = 5.065, *p* < 0.0001), and time × treatment interaction (*F*_213, 1,440_ = 6.880, *p* < 0.0001) was observed. The vehicle and coadministration dose–response curves were marked by the letters A′ and B′ for the vehicle and A for coadministration treatment to evaluate respiratory electrical parameters by plethysmography (Table 5). Baseline respiratory parameters in vehicle-treated mice did not differ from those observed in untreated mice (data not shown); however, after vehicle treatment, a physiological decrease in breathing frequency (fr_ie) of ∼5% (Table 5; significant effect of treatment, *t* = 2.579, df = 28, *p* = 0.0155) and an increase in BB of ∼5% (Table 5; significant effect of treatment, *t* = 2.482, df = 28, *p* = 0.0194) were observed. After coadministration, section A (Table 5) showed that the breathing frequency (fr_ie) increased (∼48%, Table 5; significant effect of treatment, *t* = 11.33, df = 28, *p* < 0.0001), the BB decreased (∼31%; Table 5; significant effect of treatment, *t* = 14.34, df = 28, *p* < 0.0001), and the RT decreased (∼34%; Table 5; significant effect of treatment, *t* = 7.183, df = 28, *p* < 0.0001). In addition, section B (Table 5) showed similar changes: breathing frequency (fr_ie) increased (∼28%; Table 5; significant effect of treatment, *t* = 6.321, df = 28, *p* < 0.00001), BB decreased (∼20%; Table 5; significant effect of treatment, *t* = 7.275, df = 28, *p* < 0.0001), and RT decreased (∼24%; Table 4; significant effect of treatment, *t* = 4.840, df = 28, *p* = 0.0013).

**Table 5:** Effect of systemic administration of vehicle (A′ and B′) and coadministration of (±)*cis*-4,4′-DMAR (1 mg/kg) and (±)*trans*-4,4′-DMAR (30 mg/kg) (A, B) on respiratory electrical parameters (plethysmography analysis).

Plethysmography parameters					
Vehicle					
	fr_ie	BB_ie	TV	exp/insp_V_ratio_aver	RT
BASAL	306.73 ± 4.16	196.98 ± 2.57	0.11 ± 0.002	−0.63 ± 1.54	74.65 ± 2.55
A′	292.59 ± 3.56*	206.51 ± 2.85*	0.11 ± 0.001	−0.84 ± 1.55	76.09 ± 1.43
B′	304.16 ± 2.12	198.40 ± 1.44	0.11 ± 0.002	0.17 ± 0.75	77.24 ± 1.05
(±)*cis*-4,4′-DMAR (1 mg/kg) and(±)*trans*-4,4′-DMAR (30 mg/kg)					
BASAL	301.79 ± 2.61	199.51 ± 1.77	0.12 ± 0.009	−0.03 ± 0.12	75.41 ± 0.70
A	446.87 ± 12.53***	136.56 ± 4.01***	0.11 ± 0.006	0.71 ± 4.84	49.64 ± 3.51***
B	389.02 ± 13.55***	157.93 ± 5.43***	0.11 ± 0.006	0.11 ± 4.48	56.90 ± 3.76**

Data are relative to the coadministration of (±)*cis*-4,4′-DMAR (1 mg/kg) and (±)*trans*-4,4′-DMAR (30 mg/kg) BR dose–response curve (Figure 7). Data are expressed as the mean overall effects across 15 evaluations for baseline and after vehicle or JWH-018 treatment in four mice. Data are analyzed using iox2 software and expressed as mean ± SEM of evaluations recorded in a 1-minute time window, capturing data at an acquisition frequency of 1,000 Hz. Statistical analysis was performed by Student's *t*-test for each basal and after-treatment parameter comparisons. FR_IE (bpm; breathing frequency); BB_IE (ms; breath length computed by adding inspiration and expiration duration); TV (mL/s; tidal inspiration volume); EXP/INSP_V_RATIO AVER (ratio of expired volume/tidal volume); RT (ms; relaxation time).

* *p* < 0.05.

** *p* < 0.01.

*** *p* < 0.001.

### 3.5 Morphological, histochemical, and immunohistochemical data

All reactions were conducted on sagittal cardiac tissue sections from vehicle-, (±)*cis*-4,4′-DMAR (10 mg/kg)-, (±)*cis*-4,4′-DMAR (1 mg/kg)-, and (±)*trans*-4,4′-DMAR (30 mg/kg)-treated mice, focusing on the left ventricle and vessel wall. In particular, all examinations concentrated on cardiomyocytes and the left ventricular descending artery, where morphological/histochemical alterations, oxidative stress, and inflammation are predominantly localized.

#### 3.5.1 (±)*cis*-4,4′-DMAR (10 mg/kg) and (±)*cis*-4,4′-DMAR (1 mg/kg) and (±)*trans*-4,4′-DMAR (30 mg/kg) caused significant alterations in cardiac tissue

**Figure 8:**
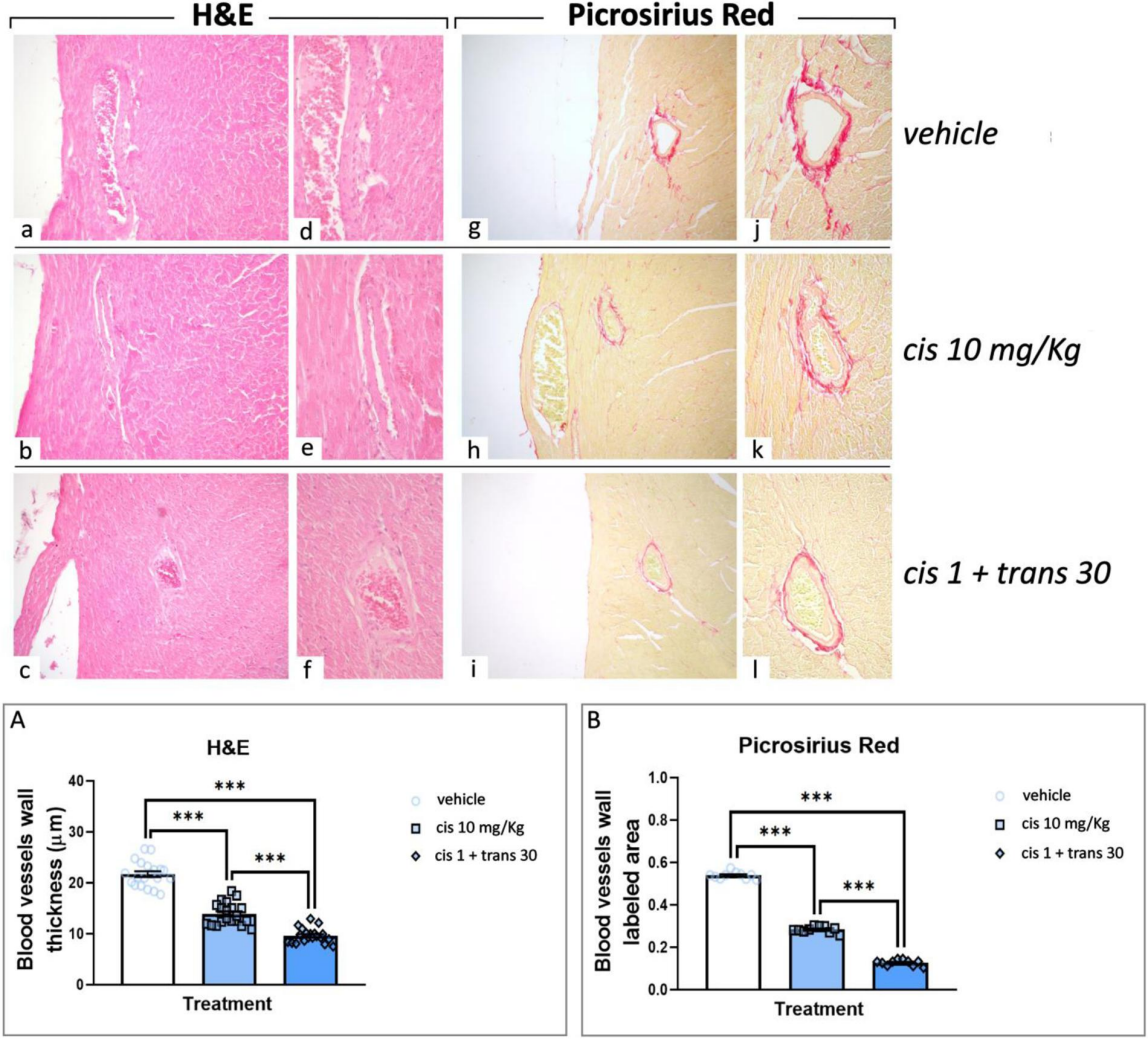
Cardiac tissue from vehicle-treated mice, mice treated with (±)*cis*-4,4′-DMAR (10 mg/kg), and mice treated with the coadministration of (±)*cis*-4,4′-DMAR (1 mg/kg) and (±)*trans*-4,4′-DMAR (30 mg/kg) stained with hematoxylin and eosin (H&E; **a**–**f**) and Picrosirius Red (PSR; **g**–**l**). Vessel wall thickness and PSR-positive area (**A**,**B**) were markedly reduced in both treatment groups compared with vehicle controls, with more pronounced changes following coadministration. Light microscopy magnifications: 20× **(a–c,g–i)** and 40× **(d–f,j–l)**. Panels **A** and **B** show quantitative analysis of vessel wall thickness and PSR-labeled area. Statistical analysis was performed using one-way ANOVA followed by Bonferroni's *post-hoc* test (***p < 0.001).

Morphological and histochemical data obtained by H&E and PSR staining in vehicle- (a–d), (±)*cis*-4,4′-DMAR (10 mg/kg)- (e–h), (±)*cis*-4,4′-DMAR (1 mg/kg), and (±)*trans*-4,4′-DMAR (30 mg/kg)-treated (i–l) mice are shown in Figure 8. Vehicle-, (±)*cis*-4,4′-DMAR (10 mg/kg)- and (±)*cis*-4,4′-DMAR (1 mg/kg) + (±)*trans*-4,4′-DMAR (30 mg/kg)-treated mice displayed a well-preserved physiological histoarchitecture of the heart, characterized by typical cardiomyocytes with acidophilic cytoplasm, centrally located nuclei, myofibrillar structure with striations, and several small blood vessels. Specifically, an extremely significant decrease in (i) arteriole wall thickness, as evidenced by H&E staining, and (ii) PSR-labeled vessel area was observed both in (±)*cis*-4,4′-DMAR (10 mg/kg)-treated mice and (±)*cis*-4,4′-DMAR (1 mg/kg) + (±)*trans*-4,4′-DMAR (30 mg/kg) coadministered mice compared with vehicle-treated mice. Notably, these observed reductions were more pronounced in mice receiving (±)*cis*-4,4′-DMAR (1 mg/kg) + (±)*trans*-4,4′-DMAR (30 mg/kg) than in mice receiving (±)*cis*-4,4′-DMAR (10 mg/kg) (*F*_(2, 57)_ = 172, *p* < 0.001 and *F*_(2, 27)_ = 1,929, *p* < 0.001 for H&E and PSR, respectively; Figure 8A,B).

#### 3.5.2 (±)*cis*-4,4′-DMAR (10 mg/kg) and (±)*cis*-4,4′-DMAR (1 mg/kg) + (±)*trans*-4,4′-DMAR (30 mg/kg) enhanced inflammation and oxidative stress to different extents

Concerning the selection of investigated markers, IL-6 was evaluated as a specific marker of inflammation (21–23), Nrf2 was assessed because it is a key factor involved in the oxidative stress cascade (24–26), and Hsp70 was studied for its cardioprotective effectiveness, playing a crucial role in hypoxic/reoxygenation/reperfusion injury of cardiomyocytes (27, 28). In addition, Hsp70 can also regulate cardiac remodeling and function in response to hypertension (29).

**Figure 9:**
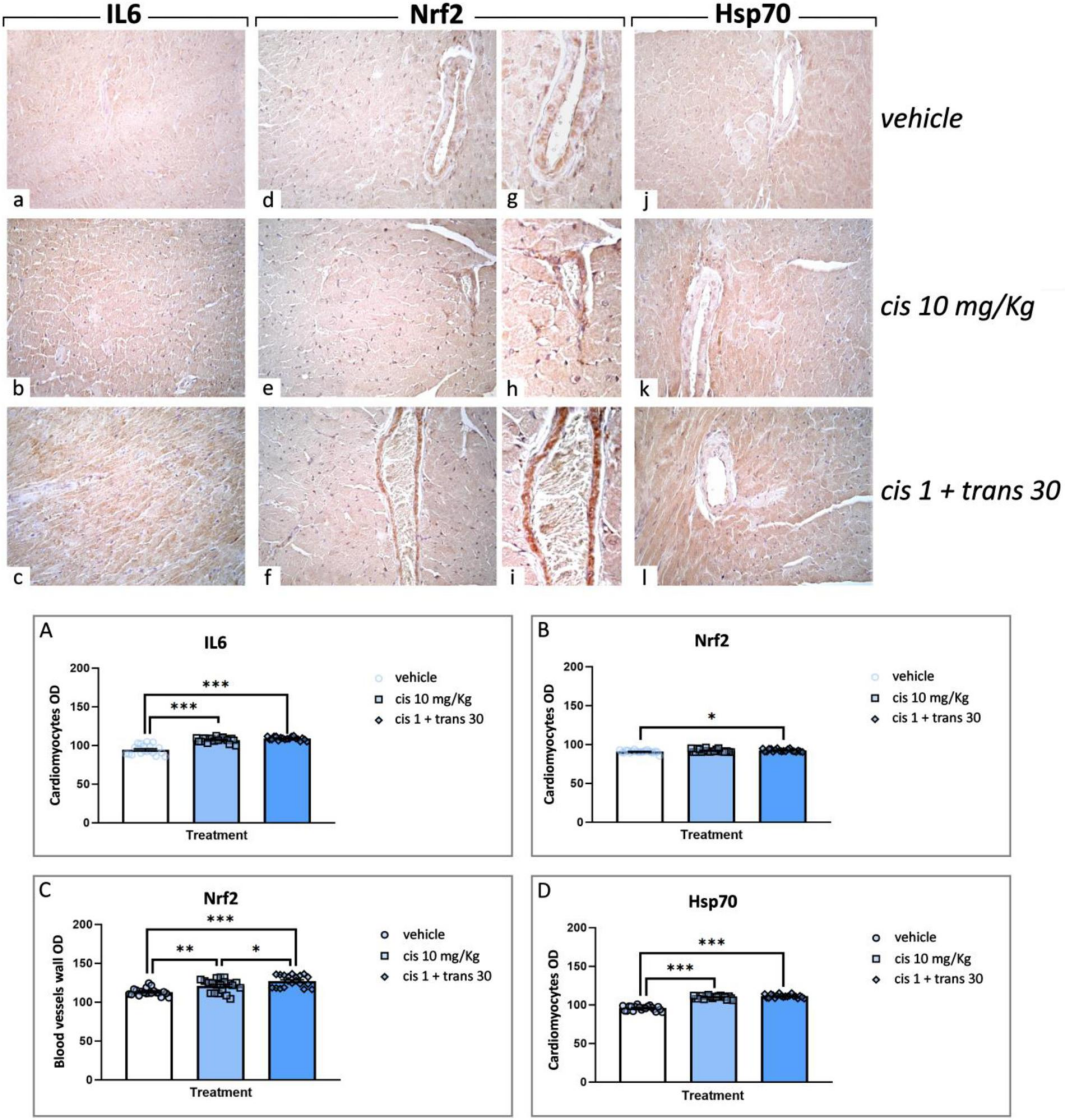
Effect of systemic administration of vehicle, (±)*cis*-4,4′-DMAR (10 mg/kg), and coadministration of (±)*cis*-4,4′-DMAR (1 mg/kg) with (±)*trans*-4,4′-DMAR (30 mg/kg) on cardiac immunostaining patterns of IL-6 **(a**–**c)**, Nrf2 **(d**–**i)**, and Hsp70 **(j**–**l)**. In both the DMAR *cis*10 group **(b)** and the group coadministered (±)*cis*-4,4′-DMAR (1 mg/kg) and (±)*trans*-4,4′-DMAR (30 mg/kg) **(c)**, cardiomyocyte IL-6 immunopositivity was noticeably greater than in vehicle-treated **(a)** mice (**A**). Regarding Nrf2, weak immunolabeling was observed in the cardiomyocytes of vehicle-treated mice **(d,g)** and DMAR *cis*10-treated animals **(e,h)**, with a significant increase after the (±)*cis*-4,4′-DMAR (1 mg/kg) and (±)*trans*-4,4′-DMAR (30 mg/kg) **(f,i)** coadministration (**B**). The strongest Nrf2 immunopositivity appeared distinctly localized in the blood vessel wall and was most pronounced in the (±)*cis*-4,4′-DMAR (1 mg/kg) and (±)*trans*-4,4′-DMAR (30 mg/kg) **(f,i)** coadministration group; Nrf expression decreased after DMAR *cis*10 **(e,h)** administration and was lowest in vehicle-treated animals **(d,g)** (**C**). In controls (i.e., vehicle animals), Hsp70 immunopositivity was manifestly stronger in cardiomyocytes of both DMAR *cis*10-treated mice **(k)** and (±)*cis*-4,4′-DMAR (1 mg/kg) and (±)*trans*-4,4′-DMAR (30 mg/kg) coadministered mice **(l)** than in vehicle-treated **(j)** animals (**D**). Light microscopy magnification: 20× **(a–f,j–l)** and 40× **(g–i)**. Panels **A–D** show quantitative analysis of IL-6, Nrf2, and Hsp70 immunopositive optical density. Statistical analysis was performed using the Kruskal–Wallis test followed by Dunn's *post-hoc* test or one-way ANOVA followed by Bonferroni's *post-hoc* test (*p < 0.05, **p < 0.01, ***p < 0.001).

Evaluation of the presence and distribution of all assessed markers after systemic administration of (±)*cis*-4,4′- DMAR and (±)*trans*-4,4′-DMAR showed extensive spread within cardiac myofibrillar cells. Notably, with regard to Nrf2, pronounced immunopositivity was also particularly evident in the tunica media of the blood vessel wall, specifically in the smooth muscle component (Figure 9). Considering IL-6 immunoreactivity, (±)*cis*-4,4′-DMAR (10 mg/kg), (±)*cis*-4,4′-DMAR (1 mg/kg), and (±)*trans*-4,4′-DMAR (30 mg/kg) significantly increased immunopositivity, as measured by the OD of cardiomyocytes, compared with vehicle-treated mice. Interestingly, no statistically significant difference in OD values was observed between the (±)*cis*-4,4′-DMAR 10 mg/kg group and the (±)*cis*-4,4′-DMAR (1 mg/kg) and (±)*trans*-4,4′-DMAR (30 mg/kg) coadministration group (*F*_(2, 56)_ = 69.7, *p* < 0.001) (Figure 8A).

Quantitative evaluation of Nrf2 immunoreactivity in cardiomyocytes revealed no significant difference in OD values between vehicle-treated mice and (±)*cis*-4,4′-DMAR (10 mg/kg)-treated animals; however, a significant increase was observed when comparing vehicle-treated mice with animals coadministered with (±)*cis*-4,4′-DMAR (1 mg/kg) and (±)*trans*-4,4′-DMAR (30 mg/kg). Notably, a slight increase was also observed in mice treated with (±)*cis*-4,4′-DMAR (1 mg/kg) and (±)*trans*-4,4′-DMAR (30 mg/kg) compared with animals treated with (±)*cis*-4,4′-DMAR (10 mg/kg) (K–W statistic=6.99, *p* = 0.03) (Figure 9B). Furthermore, analysis of tunica media of blood vessel walls revealed that the systemic administration of (±)*cis*-4,4′-DMAR 10 mg/kg or (±)*cis*-4,4′-DMAR (1 mg/kg) and (±)*trans*-4,4′-DMAR (30 mg/kg) caused a significant increase in Nrf2 expression levels compared with vehicle-treated animals. In particular, immunopositivity, measured as smooth muscle cell OD, was augmented, with the most pronounced effect observed in mice receiving the combined treatment of (±)*cis*-4,4′-DMAR (1 mg/kg) and (±)*trans*-4,4′-DMAR (30 mg/kg) (*F*_(2, 57)_ = 20.4, *p* < 0.001; Figure 9C).

Regarding Hsp70, an immunolabeling trend similar to that observed for IL-6 was detected. In fact, both (±)*cis*-4,4′-DMAR (10 mg/kg) and (±)*cis*-4,4′-DMAR (1 mg/kg) + (±)*trans*-4,4′-DMAR (30 mg/kg) significantly augmented the immunopositivity, as reflected by increased cardiomyocyte OD values. Notably, statistically significant differences were observed when estimating OD values for mice treated with (±)*cis*-4,4′-DMAR (10 mg/kg) and those receiving the combined treatment of (±)*cis*-4,4′-DMAR (1 mg/kg) and (±)*trans*-4,4′-DMAR (30 mg/kg) (*F*_(2, 57)_ = 241, *p* < 0.001; Figure 9D).

## 4 Discussion

The present study demonstrates, for the first time, cardiac and respiratory damage induced by the administration of (*±*)*cis-*4,4′-DMAR and by its coadministration with the stereoisomer (±)*trans-*4,4′-DMAR. The most severe effects were observed following administration of 30 mg/kg (*±*)*cis-*4,4′-DMAR and after coadministration of 1 mg/kg (*±*)*cis-*4,4′-DMAR and 30 mg/kg (±)*trans-*4,4′-DMAR. Although differing in intensity, these treatment regimens induce tachycardia, narrow-QRS arrhythmias, prolongation of the QT and QTcF intervals, and tachypnea, together with a decrease in RT. In addition, in a second step, which focuses on the evaluated doses and on the effects of combined administration, this investigation revealed that both (*±*)*cis-*4,4′-DMAR (10 mg/kg) and (*±*)*cis-*4,4′-DMAR (1 mg/kg) + (±)*trans-*4,4′-DMAR (30 mg/kg) triggered significant alterations in the expression of specific markers, that is, IL-6, Nrf2, and Hsp70, which were immunohistochemically investigated as key molecules crucially involved in the cardiac inflammatory/oxidative stress pathway, with Hsp70 known to possess cardioprotective effectiveness. These alterations were accompanied by histoarchitectural changes in cardiac specimens. Cardiovascular toxicity induced by synthetic stimulants includes reduced left ventricular ejection fraction, dilated cardiomyopathy, acute myocardial infarction/necrosis, arrhythmias, and ventricular hypertrophy/fibrosis (30). Despite these severe adverse effects, the most reported symptom in synthetic stimulant users is palpitations, and the most predominant HR impairment is sinus tachycardia (31), probably due to the general agitation induced by stimulant drugs and the effect of these on the monoamine transporter (32).

Similar to other synthetic stimulants, 4,4′-DMAR induced an increase in HR. Although this substance has been responsible for poisoning and death (9, 10, 12), no clinical studies have specifically reported its cardiac effects in humans. Nevertheless, based on drug forum research, users reported cardiotoxicity after 4,4′-DMAR abuse, especially hypertension and tachycardia (33, 34). These cardiovascular effects may be related to the excessive release of norepinephrine (8). *In vitro* studies conducted on HEK239 cells showed the high affinity of 4,4′-DMAR for human monoamine transporter hNET (IC_50_ = 0.50 µM and Ki = 0.27 ± 0.06 µM) (3, 4). Moreover, the ratio of effects on transporters showed slight selectivity for NET over other transporters (NET/SERT ratio: 3.50; DAT/NET ratio: 0.48) (4). Cardiac effects induced by NE release are consistent with previous studies on other well-known synthetic stimulants, such as amphetamine and cocaine, and include the presence of sinus tachycardia and ECG changes with a prolonged QTcF interval (31). Synthetic stimulants are associated with cardiac supraventricular (atrial fibrillation) and ventricular arrhythmias, such as premature ventricular beats, monomorphic or polymorphic ventricular tachycardia, and torsade de pointe (31). An increase in sympathetic tone following an increase in NE levels could lead to dysfunction of the cardiac conduction system and binding to β- and α-cardiac adrenoceptors (35). Beyond inhibition of NET, 4,4′-DMAR also inhibits SERT and DAT with micromolar affinity (Ki = 1.9 ± 0.2 and Ki = 0.53 ± 0.04, respectively) (4). In particular, *in vitro* studies demonstrated that serotonin and dopamine can trigger arrhythmias by binding 5-HT4 and D1 receptors in cardiomyocytes, respectively (36). In addition to its action on the monoamine transporter, 4,4′-DMAR could provoke arrhythmias through direct interactions with cardiac ion channels, as reported for other stimulants (31). 4,4′-DMAR can inhibit the transient outward potassium current (K_to_) and the inward rectifier potassium current (K_ir_). Although the interaction with L-type calcium channels has not been elucidated, it can be hypothesized based on evidence that similar drugs, like methamphetamine, can inhibit these ion channels (31). However, its effect on cardiac electrical parameters primarily results from potassium channel binding. This assumption is also consistent with cardiac electrical parameter data, which showed that 4,4′-DMAR induced QT prolongation. Blockade of potassium channels causes delayed ventricular depolarization, which manifests as QT interval prolongation (37). In many cases, QT interval prolongation has been reported in cocaine and methamphetamine users (38). A prolonged QT interval is associated with a higher risk of torsade de pointes, an uncommon and distinctive form of polymorphic ventricular tachycardia that can degenerate into ventricular fibrillation (39, 40). This suggests that after consumption of 4,4′-DMAR, a potential risk of torsade de pointes and triggers ventricular fibrillation exists, as has already been suggested for other NPSs (15, 41). Ventricular arrhythmias and torsade de pointes can lead to syncope and sudden cardiac death/arrest, even in otherwise healthy young individuals, highlighting the potentially life-threatening cardiotoxic effects associated with synthetic stimulant abuse (42).

Administration of (*±*)*cis-*4,4′-DMAR at the highest dose administered and the coadministration of 1 mg/kg (*±*)*cis-*4,4′-DMAR and 30 mg/kg (±)*trans-*4,4′-DMAR also induced long-lasting tachypnea, similar to observations reported for other cathinone-like stimulants (43). The tachypnea induced by 4,4′-DMAR could result from psychomotor agitation observed after administration of the comparable dosages of the drug (14). Moreover, aminorex stimulants have been associated with the development of primary arterial pulmonary hypertension (44), which includes symptoms such as fatigue, shortness of breath, cough, dizziness, chest pain, and tachypnea (45). These findings suggests that 4,4′-DMAR could impair respiratory function and decrease RT, as our results on plethysmographic parameters have shown, and may be associated with breathing weariness.

In line with these findings, subsequent histological, histochemical, and immunohistochemical examinations indicated the occurrence of cardiac injury after administration of (*±*)*cis*-4,4′-DMAR (10 mg/kg), with even pronounced alterations observed after coadministration of the lowest dose tested (1 mg/kg) and its (*±*)*trans* stereoisomer (30 mg/kg). In particular, significant changes were detected in the expression of specific immunohistochemical markers, accompanied by the presence of histoarchitectural alterations, such as a significant decrease in arteriolar wall thickness and PSR-labeled vessel area. Based on previous literature describing the cardiotoxic effects of other stimulant drugs (e.g., MDMA, cocaine) and recreational drugs, including new psychoactive substances (e.g., JWH-018, α-PVP, and MDPV) (14, 15, 31, 46), we assumed that activation of inflammatory and oxidative stress pathways plays a crucial role in murine cardiovascular injury after 4,4′-DMAR administration.

Hence, we investigated the expression of IL-6, a proinflammatory, pleiotropic cytokine whose elevated levels are known to contribute to the progression of myocardial damage and dysfunction in heart failure syndrome resulting from various causes, including toxidromes (21–23). Moreover, the complex interplay between inflammatory response and oxidative stress pathways should be taken into account. Although inflammation can induce oxidative stress, it is also one of the most common outcomes of oxidative stress. Indeed, elevated oxidative stress levels might stimulate chemokine and cytokine expression, leading to increased inflammation, a typical outcome of many cardiovascular diseases, often accompanied by reduced cellular antioxidant capacity (41). Therefore, to assess the onset/enhancement of 4,4′-DMAR-induced oxidative stress, we explored a pleiotropic transcription factor, Nrf2, a key component in antioxidant defenses in cardiovascular disorders (e.g., atherosclerosis, hypertension, and heart failure), which is also crucially involved in cardioprotection against oxidative damage during ischemia–reperfusion injury processes (24–26). In addition, it is well known that toxidromes may frequently induce hyperthermic reactions (15), which in turn trigger the synthesis of Hsp70. Recent data have reported hyperthermia in 4-4′-DMAR-treated mice, accompanied by an imbalance in the oxidative state in nervous tissue (14). We assessed Hsp70 as a crucial player in cardioprotective mechanisms, hypoxic/reoxygenation injury in cardiomyocytes (27, 28), and the regulation of cardiac remodeling and function in hypertensive syndromes (29).

Our immunohistochemical data demonstrated that both (*±*)*cis*-4,4′-DMAR (10 mg/kg) and the coadministration of (±)*cis*-4,4′-DMAR (1 mg/kg) and (±)*trans*-4,4′-DMAR (30 mg/kg) led to a marked overexpression of IL-6 and Hsp70 in cardiomyocytes, without any difference in the extent of immunolabeling. In contrast, the coadministration of (±)*cis*-4,4′-DMAR (1 mg/kg) and (±)*trans*-4,4′-DMAR (30 mg/kg) triggered an increase in Nrf2 expression levels in cardiomyocytes; however, notably, both (±)*cis*-4,4′-DMAR 10 mg/kg alone and the coadministration of (±)*cis*-4,4′-DMAR (1 mg/kg) and (±)*trans*-4,4′-DMAR (30 mg/kg) caused a striking increase in Nrf2 in the smooth muscle cells of the blood vessel wall.

Concerning both IL-6 and Nrf2, experimental and epidemiological studies have revealed a potential dichotomous role for these two molecules, which can attenuate or, contrarily, intensify heart failure and cardiovascular syndromes (47). In fact, studies on myocardial infarction have established how short-term IL-6 signaling protects and preserves cardiac tissue in response to acute injury, while IL-6 overproduction plays a causal role in cardiovascular disease. Highlighting a similar dual role, evidence suggests that Nrf2 activity may lead to beneficial or even detrimental effects in cardiovascular syndromes (24–26).

Based on our current data, we may hypothesize that the elevated IL-6 levels may lead to an imbalance in the oxidative state, leading to ROS overexpression, which in turn triggers the activation of the oxidative stress pathway. Consequently, a recovery mechanism may be initiated, with Nrf2 and Hsp70 acting as key factors. Specifically, based on their “varying faces,” the increased expression of Nrf2 and Hsp70, characterized by a diverse extent across different cardiac tissue compartments, could represent both the injury response and a counteracting strategy to repair or at least reduce tissue damage.

In conclusion, our results highlights, for the first time, the pronounced cardiotoxic effects of both (*±*)*cis-*4,4′-DMAR at the highest dose tested (10 mg/kg) and the coadministration of the lowest dose tested (1 mg/kg) of (*±*)*cis-*4,4′-DMAR with its (±)*trans* stereoisomer (30 mg/kg). We confirmed a preliminary neurobehavioral, physiological, and immunohistochemical study (14), as well as a genotoxicology study (48), and showed that (*±*)*cis-*4,4′-DMAR had greater effects on cardiac function than its stereoisomer (±)*trans-*4,4′-DMAR. Nevertheless, the coadministration of 1 mg/kg of (*±*)*cis-*4,4′-DMAR and 30 mg/kg of (±)*trans-*4,4′-DMAR, which were ineffective if administered alone, induced severe cardiac adverse effects, suggesting that the (±)*trans* stereoisomer could potentiate the effect of (*±*)*cis* stereoisomer (14). This evidence is probably due to differences in the metabolism of these stereoisomers (49). Indeed, as Tirri and colleagues proposed in excretion studies, the stereoisomer (±)*trans* could reduce the metabolism of the (*±*)*cis* form, thus increasing the plasma level of the substance and, consequently, its potency (14). Our results on the cardiotoxicity of both the stereoisomer (*±*)*cis-*4,4′-DMAR and its coadministration with the (±)*trans* stereoisomer (30 mg/kg) represent a step forward in understanding the dramatic effects of NPSs, and future studies are welcome to identify how to counteract these effects.

## Data Availability

The raw data supporting the conclusions of this article will be made available by the authors, upon reasonable request.
